# Correlation of size specific dose estimates with organ dose in abdominal spectral computed tomography

**DOI:** 10.1002/acm2.70845

**Published:** 2026-10-05

**Authors:** Tian Qin, Qingting Sun, Yihan Fan, Mengting Wang, Jing Wang, Yankui Chang, Baohui Liang

**Affiliations:** ^1^ Division of Life Sciences and Medicine The First Affiliated Hospital of USTC, University of Science and Technology of China Hefei Anhui China; ^2^ School of Medical Imaging Bengbu Medical University Bengbu Anhui China; ^3^ Department of Oncology The Second Hospital of Anhui Medical University Hefei Anhui China; ^4^ Department of Radiology The First Affiliated Hospital of Bengbu Medical University Bengbu Anhui China; ^5^ Anhui Key Laboratory of Digital Medicine and Intelligent Health Bengbu Medical University Bengbu Anhui China

**Keywords:** computed tomography, Monte Carlo, organ dose, size‐specific dose estimate, spectral CT

## Abstract

**Background:**

In abdominal spectral Computed Tomography (CT), organ dose assessment is important for radiation safety but remains challenging due to the complexity of Monte Carlo (MC) simulations.

**Purpose:**

This study aimed to compare the correlations of three size‐specific dose estimate (SSDE) metrics (including the central slice SSDE_center_, the mean of all slices SSDE_mean_, and the organ‐specific SSDE_organ_) with organ doses and to establish a rapid estimation method for organ dose using linear regression in abdominal spectral CT.

**Methods:**

Monte Carlo N‐Particle (MCNP6) was used to model the General Electric (GE) Revolution CT scanner, including its bowtie filter, radiation source, and simulations of tube voltage switching and the X‐ray tube trajectory. Digital Imaging and Communications in Medicine (DICOM) data from 30 patients undergoing abdominal spectral CT with identical scan parameters were processed using 3D Slicer. Radiation‐sensitive organs were delineated. Following this, digital models were constructed and subsequently voxelized. All patient external body contours were delineated using ImageJ software. Three SSDE metrics were calculated for all patients. Linear regression analysis was performed to correlate organ doses with the three SSDE metrics and to derive estimation coefficients.

**Results:**

The results indicated that in abdominal spectral CT examinations on a Revolution scanner, SSDE_organ_ exhibited the strongest correlation with organ dose. The R^2^ values for the liver, stomach, pancreas, spleen, kidneys, spinal cord, and bladder were 0.89, 0.61, 0.81, 0.85, 0.88, 0.94, and 0.68, respectively. The corresponding estimation coefficients derived from SSDE_organ_ for these organs were 0.42, 0.36, 0.37, 0.41, 0.36, 0.26, and 0.26, respectively. The estimation errors for the conversion coefficient combined with SSDE_organ_, relative to the MC simulation results, were 7.34% for the liver, 6.09% for the kidneys, 13.25% for the spleen, 2.97% for the spinal cord, 13.61% for the pancreas, 17.64% for the stomach, and 18.56% for the bladder.

**Conclusions:**

For abdominal spectral CT, SSDE_organ_ demonstrated the strongest correlation with individual organ doses. These findings enable rapid and accurate estimation of organ dose, providing a basis for personalized radiation dose assessment in clinical practice.

## 1 INTRODUCTION

Spectral CT, implemented via rapid kV‐switching between high and low tube voltages on the GE Revolution CT system, enables simultaneous acquisition of projection data at two distinct energy levels.^1^ This technique offers capabilities unavailable in conventional single‐energy CT (SECT), including virtual monoenergetic imaging, material decomposition, spectral curve analysis, and effective atomic number determination, thereby substantially enhancing the clinical diagnostic value of abdominal examinations.^2^, ^3^ However, the use of multiple energy spectra renders radiation dosimetry more complex compared with conventional SECT. Consequently, organ dose assessment for spectral CT examinations is essential to ensure an appropriate balance between radiation dose, patient safety, and image quality.^4^ Due to the varying radiation sensitivity of different organs, organ dose is commonly used in clinical examinations to characterize the radiation dose received by each specific organ. One method for assessing organ dose is the direct physical measurement of an individual organ's dose using dosimetric tools. This is typically performed using small in vivo dosimeters, namely thermoluminescent dosimeters (TLD) and metal‐oxide semiconductor field‐effect transistor (MOSFET) detectors. These dosimeters are highly energy‐dependent, making calibration in a dual‐energy environment more challenging; furthermore, the in vivo measurement approach is not feasible for clinical application.^5^, ^6^, ^7^ Monte Carlo (MC) simulation software (such as Monte Carlo N‐Particle, MCNP) is a widely used method for CT organ dose calculation and has been employed by numerous researchers in dosimetric studies of conventional CT.^8^, ^9^, ^10^, ^11^, ^12^ Currently, MC simulations have also been applied in some dosimetric studies of spectral CT (such as organ dose assessment or derivation of dose conversion factors).^13^, ^14^, ^15^ However, the establishment of an MC model suitable for comprehensive abdominal organ dose evaluation for the rapid kV‐switching spectral CT mode employed on the GE Revolution platform remains insufficiently explored. Furthermore, the MC method itself is computationally complex and time‐consuming, making it difficult to implement in routine clinical practice. Previous studies have demonstrated that in conventional SECT, the size‐specific dose estimate (SSDE) incorporating patient‐specific size information exhibits a stronger correlation with organ dose compared to the volume CT dose index (CTDI_vol_).^16^, ^17^ However, multiple methods exist for calculating SSDE itself, such as organ‐specific SSDE (SSDE_organ_), SSDE based on water equivalent diameter (WED) in the mean of the scan range (SSDE_mean_), and SSDE based on WED in the center of the scan range (SSDE_center_).^18^, ^19^, ^20^ It remains unclear how the correlation strengths of these different SSDE methods with organ dose compare in the context of spectral CT scanning. SSDE has previously been discussed in the context of spectral CT, for example, in analyses of the influence of the X‐ray spectrum on its calculation,^21^ comparisons of image quality and overall radiation dose between dual‐energy and SECT,^22^ and in clinical reviews mentioning dose considerations.^23^ However, for abdominal rapid kV‐switching spectral CT, there is still a lack of research systematically investigating the correlation between SSDE_organ_, SSDE_mean_, SSDE_center_, and organ‐specific doses obtained from MC simulations.

Therefore, this study plans to utilize MCNP6 software to establish an MC model for the rapid kV‐switching spectral CT mode of the GE Revolution scanner and to simulate doses to radiation‐sensitive abdominal organs. The primary objectives are: (1) to determine which of the three SSDE metrics correlates most strongly with actual organ doses under this specific scanning mode; and (2) to establish a practical linear regression model to enable rapid and accurate estimation of individual organ doses based on clinically available SSDE data. This approach aims to bridge the gap between time‐consuming MC simulations and the clinical need for individualized radiation dose assessment in abdominal spectroscopy CT examinations.

## 2 MATERIALS AND METHODS

### 2.1 Specifications of the spectral CT scanner

MCNP6 software was used to create a Monte Carlo model of the GE Revolution CT scanner (GE Healthcare, Waukesha, Wis). This system utilizes rapid kV‐switching at the tube to enable dual‐energy X‐ray output from a single source. Tube current modulation (TCM) is disabled during spectral scanning.

This study retrospectively collected imaging data from 30 patients (11 male, 19 female; mean age 58.9 ± 12.3 years, range 32–74 years) who underwent full abdominal spectral CT scans between January 2024 and July 2024. The patients' body sizes, characterized by WED, varied considerably, with a mean of 24.6 ± 3.0 cm (range: 17.3–29.5 cm). The specific scanning parameters are provided in Table 1.

**TABLE 1:** Scanning parameters for abdominal spectral CT.

Scanning parameters of the revolution CT	
Tube Voltage	Rapid kV‐Switching (80/140 kV) with single tube
Rotation Time	0.5 s
Temporal Resolution	0.5 ms
Beam Collimation	80 mm
Pitch	0.992
Scan Field of View	Large Body
Field of View	50 cm
Tube Current	405 mA
CTDI_vol_	10.1 mGy

Because TCM was disabled, all 30 patients were scanned with the identical fixed tube current (405 mA) and CTDI_vol_ (10.1 mGy). Accordingly, the same dose normalization factor was applied to all patient‐specific MC simulations, and variations in organ doses among patients were attributable primarily to differences in patient body size (i.e., WED and anatomical geometry).

This retrospective study was approved by the Ethics Committee of Bengbu Medical University (NO.2025‐508). The requirement for written informed consent was waived by the IRB due to the retrospective nature of the study and the use of anonymized patient imaging data.

### 2.2 Construction of voxelized patient models

Quantifying the radiation dose delivered to various organs of patients is crucial for ensuring radiation protection. Currently, the preferred method involves voxelizing patient CT images using software, followed by MC simulation, substituting for the impractical direct dosimetric measurements. For each of the 30 selected patients, the body, lungs, liver, pancreas, spleen, stomach, kidneys, bone, spinal cord, and bladder were delineated. The following steps were then used to create a digital voxel model for each individual: (1) The patient's Digital Imaging and Communications in Medicine (DICOM) images (specifically, the virtual non‐contrast (VNC) series generated by the Revolution CT scanner's spectral reconstruction) were imported into 3D Slicer software (https://www.slicer.org/), and each organ was delineated separately. The overall external body contour and lungs were outlined using automatic contouring (based on the VNC image HU values), bones were outlined by setting the Hounsfield unit (HU) value threshold range, and all other organs were segmented manually. (2) The segmentation information was saved in Digital Imaging and Communications in Medicine for Radiation Therapy (DICOM‐RT) format. (3) The segmentation information was imported into a GUI program written in MATLAB (The MathWorks Inc., Natick, MA, USA) for voxelization. The elemental composition and physical density of each organ were referenced from the values provided in the International Commission on Radiological Protection (ICRP) Publication 110.^24^

It should be noted that, in this study, the delineation of all organs was strictly confined to the CT slice images within the axial scan coverage. Any organ tissues extending beyond the scan range, such as the cranial and caudal extensions of the spinal cord, were excluded from both the segmentation process and the subsequent MC dose calculations. Therefore, the organ doses reported herein represent, in effect, the local mean absorbed dose to the portion of the organ exposed within the scanned field, rather than the whole‑organ mean dose for the entire organ.

### 2.3 Construction of the MC model for the revolution CT system

The MC modeling of the CT scanner primarily comprised the following components: the equivalent X‐ray energy spectrum, bowtie filter, and the X‐ray source model. In this experiment, the CT source model and bowtie filter were constructed using the method developed by Turner et al.,^25^ which includes the generation of equivalent energy spectra by the half‐value layer (HVL) method and the construction of bowtie filters by the error of weighted CT dose index (CTDI_w_). The X‐ray source model was developed with reference to the simplified modeling approaches of Gu and Pan et al.^8^, ^26^

#### 2.3.1 Modeling of the X‐ray energy spectrum

The equivalent energy spectrum was constructed with reference to the method described by Turner et al.^25^ In this study, a pencil ionization chamber and aluminum sheets of varying thicknesses were used to measure the first HVL of X‐rays at different tube voltages. The obtained HVL values were then input into MATLAB with the integrated SPEKTR 3.0 toolkit to generate the corresponding equivalent energy spectrum.^27^ The validity of these spectra was confirmed by comparing the measured HVL with the HVL output by SPEKTR 3.0.

#### 2.3.2 Modeling of the bowtie filter

In this study, following the equivalent geometric simplification approach proposed by Turner et al.,^25^ we did not perform additional physical dose profile measurements. Instead, we modeled a rectangular structure with an ellipsoid removed to approximate the clinical body bowtie filter (only this single filter was used for the abdominal scan protocol). The geometric parameters of the filter, including the semimajor and semiminor axes of the ellipse, the rectangular height, and the embedding depth of the ellipse, were iteratively adjusted to achieve the best simultaneous match between the simulated CTDI_100_,c at the center and the average CTDI_100_,p at four peripheral positions and the corresponding measured values. Given that this study focused on adult abdominal patients, the validation phantom was the standard CTDI body phantom with a diameter of 32 cm, conforming to the abdominal phantom specification. The final geometric parameters ensured that the simulation errors for both the central and peripheral CTDI_100_ values were within acceptable ranges (see Supplementary Materials), thereby confirming the overall equivalence of the equivalent filter in terms of beam hardening effects.

#### 2.3.3 MC modeling of the revolution CT spectral scan mode

Based on the ‘dose‐equivalent’ concept, a defined number of X‐ray sources were placed at a specific distance from the isocenter to simulate a single‐slice axial scan. In this study, this distance was set to 62.56 cm (corresponding to the source's distance from the isocenter in the Revolution CT user manual). It is important to note that while increasing the number of simulated X‐ray sources improves result accuracy, it also proportionally increases simulation time, necessitating a balance between the number of sources and the accuracy of the simulation. Khursheed et al.,^28^ demonstrated that simulating 18 source angles per rotation yielded CTDI results with minimal difference compared to using 72 angles, deeming 18 angles sufficient to model a full tube rotation. Consequently, to maintain acceptable model error, this study ultimately employed 36 sources arranged around the patient—18 assigned to each of the two tube voltages, positioned in an alternating pattern.

To model the spectral scanning mode of the Revolution CT, a clear grasp of its working mechanism is essential. The principles of rapid kV‐switching spectral CT have been extensively detailed in prior literature.^29^, ^30^, ^31^ In simple terms, when the Revolution CT performs a spectral scan, the tube voltage rapidly switches between 80 and 140 kV, ensuring sufficient spectral separation between the X‐ray beams at these two energies, with a switching cycle of 0.5 ms (meaning each of 80 and 140 kV lasts for 0.25 ms. The principle is illustrated in Figure 1a. This scanning method enables spectral analysis in the projection data space and performs CT reconstruction in the raw data domain.^32^, ^33^ Consequently, constructing the MC model for the Revolution CT's spectral mode requires a two‐step process. The 360° distributed source was divided into two parts for MC simulation. Each part was assigned a specific tube voltage: Model 1 (18 sources and 18 bowtie filters) was designated for 80 kV, as illustrated in Figure 1b, while Model 2 (18 sources and 18 bowtie filters) was designated for 140 kV, as shown in Figure 1c. The results of these simulations were subsequently aggregated to formulate what is referred to as the equivalent 360° source model, which serves to approximate the actual 360°CT scan. Based on the above principles, each slice in the MC simulation requires two separate simulation calculations for the same tomographic slice.

**FIGURE 1:**
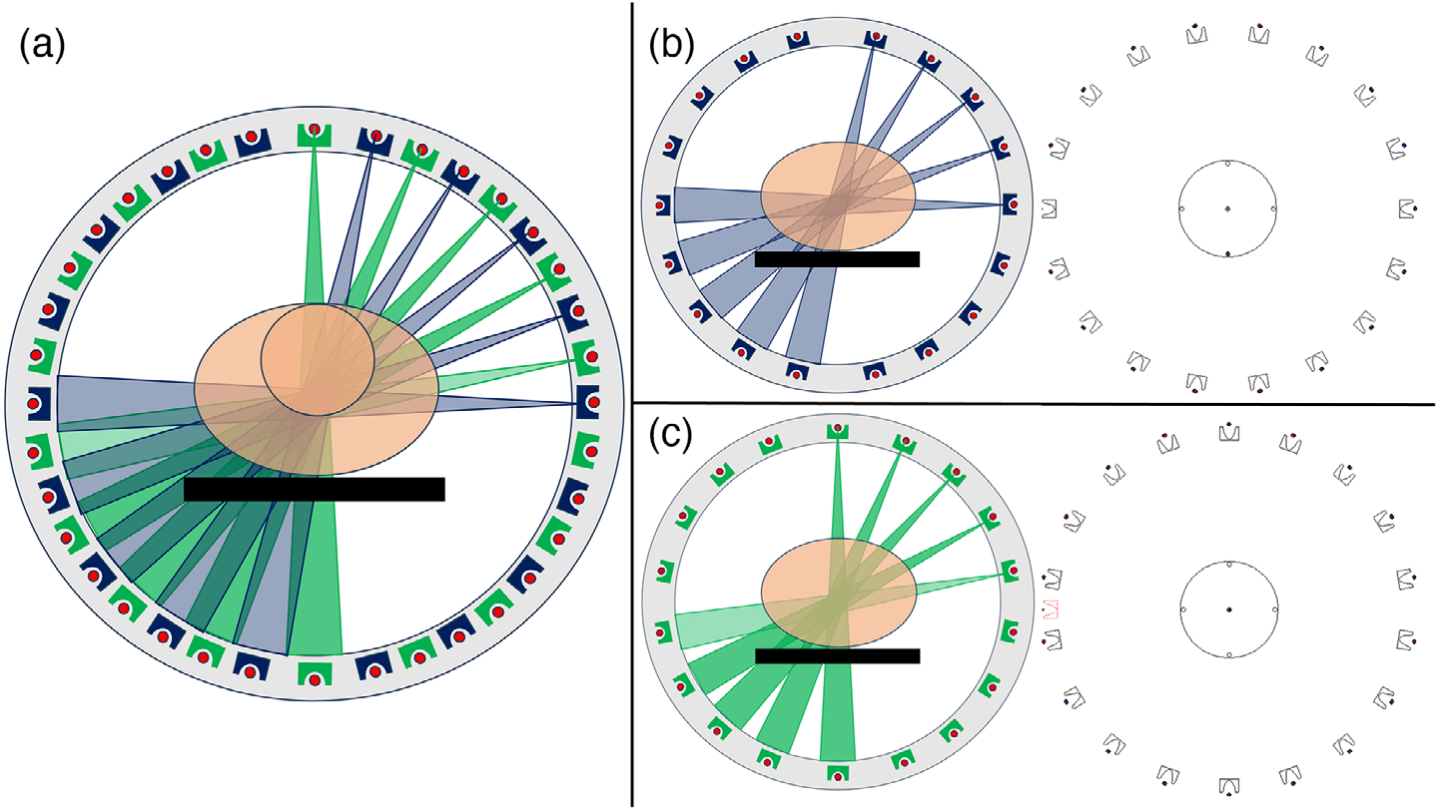
Schematic of the revolution spectral CT principle and its MC modeling. (a) Schematic of the Revolution CT spectral scan principle, where green and blue denote X‐rays of two different energies. (b, c) Schematics of the independent MC models established for each of the two X‐ray energy levels.

### 2.4 Validation of CT scanner models

For the X‐ray energy range used in CT examinations, the interaction of photons with human tissues involves both the photoelectric effect and Compton scattering. The angular distribution of the secondary electrons produced by these interactions is energy‐dependent; it is broader at lower energies. The energy is deposited at the location where they interact with the photon. When secondary electrons ionized from superficial tissues travel to their maximum range, charged‐particle equilibrium (CPE) can be achieved at that location.^11^ Given that the energy lost by secondary electrons to bremsstrahlung radiation is minimal, the kerma can be approximated as equivalent to the organ's absorbed dose in this scenario. In this study, the F6 tally, a standard MCNP cell tally that scores collision kerma (energy deposition per unit mass) within a cell, was employed to record energy deposition. The F6 tally normalizes the simulation output per source particle in units of MeV/gram/ particle. The conversion of the units is carried out utilizing a conversion factor (CF), which is calculated as follows:

(1) $$\begin{array}{c} CF=\frac{K_{air,M}}{K_{air,S}}\; \end{array}\;$$

Where $K_{air,\;M}$ is the air kerma measured in situ at the gantry isocenter using a pencil ionization chamber (RaySafe Xi; RaySafe AB Corporation, Billdal, Sweden) (mGy/100mAs), and $K_{air,\;S}$ is the air kerma simulated within the equivalent pencil ionization chamber model constructed in MCNP under the same scan protocol (MeV/gram/per particle). Based on the geometric and physical specifications provided in the pencil ionization chamber's manual, a corresponding model was built within the MCNP framework.

During the actual measurement, a pencil ionization chamber was used to measure the dose values at each hole position in the CTDI phantom. Except for the hole occupied by the ionization chamber, all other holes were plugged with polymethylmethacrylate (PMMA) rods of the same material as the phantom. The radiation dose values from five measurement positions (the center hole and four peripheral holes of the CTDI phantom) were measured, and the CTDI_w, M_ was subsequently calculated. For MC simulations, a CTDI phantom with a diameter of 32 cm and a length of 15 cm was constructed. Following the same procedure as the experimental measurement, the simulations were performed to calculate CTDI_100_ at the center and periphery of the CTDI phantom, from which the CTDI_w, S_ was then derived. These validation measurements were performed under both 40 and 80 mm collimations to test the MC model's accuracy, whereas both the patient data acquisition and the subsequent organ dose simulations were performed at 80 mm collimation. The above validation was based on CTDI_w_ measurements under each monoenergetic mode, which indirectly supported the simulation accuracy for the spectral scanning mode through the linear superposition principle (see Section 2.5.2). However, direct experimental validation under the spectral mode is still lacking in this study.

### 2.5 Calculation of radiation dose metrics for patients undergoing abdominal spectral CT

#### 2.5.1 Calculation of SSDE

SSDE, a patient‐specific dose metric based on WED, was introduced to address the limitations of the scanner‐reported CTDI_vol_ by incorporating individual patient size information.^16^, ^17^, ^21^ In subsequent studies, the classification of SSDE was further refined, and three SSDE metrics were proposed. SSDE_organ_, defined as the average SSDE of the slices encompassing the organ, has been shown to provide a better estimation of patient organ doses in routine CT examinations.^19^ SSDE_center_, defined as the SSDE at the central slice of the patient image series, serves as a surrogate for rapidly characterizing the overall SSDE level in abdominal scans without the need for contouring on every slice.^18^ SSDE_mean_, defined as the average SSDE across all image slices within the scan range, offers a more comprehensive calculation but is relatively time‑consuming.^20^ Although existing research suggests that SSDE_organ_ provides superior organ dose estimation in conventional CT,^19^ the performance differences among various SSDE characterization methods in spectral CT (particularly rapid kV‐switching spectral CT) remain unclear. Therefore, this study aims to systematically compare the correlations of SSDE_organ_, SSDE_mean_, and SSDE_center_ under this scanning mode to identify the optimal method for organ dose estimation. Therefore, this study aims to systematically compare the correlations of SSDE_organ_, SSDE_mean_, and SSDE_center_ under this scanning mode to identify the optimal method for organ dose estimation.

Following the methodology prescribed in AAPM Report 220, the three dose metrics were calculated based on the WED. All slice images (the VNC image series) of 30 patients were imported using ImageJ software (National Institutes of Health, Bethesda, MD, USA), and the region of interest (ROI) was outlined. Taking a single patient as an example: after loading all the slice images, the external contour of the patient was outlined as the ROI using ImageJ's built‐in threshold segmentation function; the area of the ROI and the mean of the HU values were measured and recorded. Subsequently, the WED was calculated according to Equation (2). Based on the WED, the body size correction factor f^B32^ and the SSDE were computed using Equations (3) and (4), respectively. The equations are as follows:

(2) $$\mathrm{WED}=2\sqrt{\left[\frac{1}{1000}\mathrm{HU}{\left(x,y\right)}_{\mathrm{ROI}}+1\right]\frac{A_{\mathrm{ROI}}}{\pi }},$$

(3) $$\begin{array}{c} \;f^{B32}=\alpha \times e^{-\beta *WED}, \end{array}$$

(4) $$\begin{array}{c} \mathrm{SSDE}=f^{B32}\;\times \mathrm{CTD}I_{\mathrm{vol}}, \end{array}$$

where HU(x, y)_ROI_ is the average HU value in the ROI, and A_ROI_ is the area of the ROI. Of the constants given in Report 220: α = 1.9852, β = 0.486.

The calculation of SSDE_organ_ first requires determining the organ‐specific WED (WED_organ_). WED_organ_ was defined as the average WED of the slice images corresponding to the z‐axis position of each organ. The organ‐specific SSDE is then obtained by multiplying the patient's CTDI_vol_ by the size correction factor f^B32^ specific to that organ's WED, as shown in Equations (5) and (6).

(5) $$\mathrm{WE}D_{\mathrm{organ}}=\frac{1}{k}\;\sum_{n}^{n+k}2\sqrt{\left[\frac{1}{1000}\mathrm{HU}{\left(x,y\right)}_{\mathrm{ROI}}+1\right]\frac{A_{\mathrm{ROI}}}{\pi }},$$

(6) $$\begin{array}{c} \;\;\mathrm{SSD}E_{\mathrm{organ}}=\mathrm{CTD}I_{\mathrm{vol}}\;\times \;f_{\mathrm{organ}}^{B32}, \end{array}$$

Where *n* denotes the first slice in which the organ appears, and *k* represents the total number of slices containing the organ.

The calculation of SSDE_mean_ requires determining the WED for all tomographic images, computing the SSDE_z_ for each slice, and then averaging these values. In contrast, SSDE_center_ is defined as the SSDE_z_ of the central slice in the patient's image series. The calculations for these two SSDE variants are shown in Equations (7) and (8), respectively:

(7) $$\begin{array}{c} \mathrm{SSDEz}=f_{Z}^{B32}\;\times \mathrm{CTD}I_{\mathrm{vol}}, \end{array}$$

(8) $$\mathrm{SSD}E_{\mathrm{mean}}=\frac{1}{m}\;\sum_{1}^{m}\mathrm{SSD}E_{z},$$

where $f_{Z}^{B32}$ is the $f^{B32}$ for a single slice, and m is the total number of slices in the individual patient's image series.

#### 2.5.2 Calculation of patient organ dose

The organ doses for patients during spectral CT scanning were simulated using the two‐part spectral scanning source model. The results from both parts were then summed to obtain the total dose for each organ. The GE Revolution CT user manual indicates that the two tube voltage levels are sustained for an equal duration (50:50) during switching, thereby equally splitting the total exposure. Based on the principle of rapid kV‐switching spectral scanning, the mAs for each of the two tube voltages were calculated separately.

Since the output of the MCNP simulation is not in units of mGy, a CF is required to convert the results from MeV/gram/particle to mGy/100 mAs. The calculation formula is as follows:

(9) $$D_{\mathrm{Ti}1}=D_{s1}\;\times \mathrm{CF},$$

(10) $$\begin{array}{c} \;D_{\mathrm{Ti}2}=D_{s2}\;\times \mathrm{CF}, \end{array}$$

(11) $$D_{T}=\sum_{i=1}^{k}\left[\frac{I_{i}}{100}(D_{\mathrm{Ti}1}\times +D_{\mathrm{Ti}2)}\right],$$

D_s1_ and D_s2_ are the MC simulation results for each organ on each slice at 80 and 140 kV, respectively. D_Ti1_ and D_Ti2_ are each multiplied by half of the mAs per slice, and the sum of these two products yields the total radiation dose (D_T_) for each organ in that slice. These calculated organ doses from the validated MC simulations were subsequently utilized as the reference standards (ground truths) for the linear regression analyses against the three SSDE metrics.

### 2.6 Statistical analysis

Statistical analysis was performed using Origin 2022 (OriginLab Corp., Northampton, MA, USA). Linear regression analysis was applied to fit the correlation between organ dose and the three SSDE metrics (SSDE_organ_, SSDE_mean_, and SSDE_center_). The determination coefficient (*R* ^2^) was also considered an important parameter to assess the degree of correlation between the two dose metrics, with higher values of *R* ^2^ indicating a higher degree of linear correlation between the two variables. Linear regression equations were used to fit the mathematical relationship between the two dose metrics, enabling the estimation of organ dose.

## 3 RESULTS

### 3.1 CF for abdominal spectral CT examination

The CFs for different tube voltages under 40 and 80 mm collimation are presented in Table 2.

**TABLE 2:** Conversion factors and CTDIw validation errors in abdominal spectral CT.

Tube voltage (kV)	Collimation (mm)	$K_{air,M}$ (mGy/100 mAs)	$K_{air,S}$ (MeV/gram/particle)	CF (mGy · gram · per particle /100 mAs/MeV)	CTDI_w, M_ (mGy/100 mAs)	CTDI_w, S_ (mGy/100 mAs)	Errors (%)
80	40	6.75	1.69E‐05	3.99E+05	2.12	1.96	−7.55%
	80	6.43	1.81E‐05	3.56E+05	1.88	1.75	−6.91%
100	40	12.09	1.74E‐05	6.93E+05	4.12	3.87	−6.07%
	80	11.01	1.75E‐05	6.30E+05	3.71	3.51	−5.39%
120	40	18.49	1.78E‐05	1.04E+06	6.67	6.30	−5.55%
	80	17.23	1.83E‐05	9.41E+05	5.96	5.70	−4.36%
140	40	26.04	1.86E‐05	1.40E+06	9.53	9.06	−4.93%
	80	23.10	1.84E‐05	1.25E+06	8.51	8.11	−4.70%

*Note*: Detailed comparisons between the central and peripheral measurement points are provided in Table S1 in the Supplementary Materials.

Abbreviations: CF, conversion factor; CTDI_w, M_, measured weighted CTDI (mGy/100 mAs); CTDI_w, S_, Monte Carlo simulated weighted CTDI (mGy/100 mAs); Error, (CTDI_w, M_—CTDI_w, S_) / CTDI_w, M_ × 100%; $K_{air,M}$, measured air kerma at isocenter (mGy/100 mAs); $K_{air,M}$/$K_{air,S}$, mGy∙gram∙per particle/100 mAs/MeV; $K_{air,S}$, simulated Monte Carlo value in air under the same scan protocol (MeV/gram/per particle).

### 3.2 Verification of the CT source model

The CTDI_w_ values obtained from MC simulations were multiplied by the CFs, and the resulting values were compared with the experimentally measured CTDI_w_. The results are presented in Table 2. They show that the overall error between the MC‐simulated and measured CTDI_w_ is less than 10%. The maximum error was −7.55% (80 kV, 40 mm collimation), and the minimum error was −4.36% (120 kV, 80 mm collimation). In addition, we compared the simulated and measured data for the central and peripheral holes of the CTDI phantom. The results showed that the errors at all measurement points were consistently in the same direction (with simulated values slightly lower than measured values), and there was no instance where the errors at the center and periphery were in opposite directions, which could have led to mutual cancellation after weighted summation. Detailed data are provided in Table S1 in the Supplementary Materials.

### 3.3 Linear regression analysis between radiation dose metrics and organ doses in abdominal spectral CT

Using the validated MC‐simulated organ doses (as detailed in Section 2.5.2) as the reference ground truth, the linear regression analysis revealed that these organ doses exhibited the highest correlation with SSDE_organ_. Except for the bladder and stomach, the coefficients of determination (*R* ^2^) values between organ dose and SSDE_organ_ exceeded 0.80 for all other organs, with the spinal cord showing the highest correlation (*R* ^2^ = 0.94). The correlation between organ doses and SSDE_mean_ was moderate, with *R* ^2^ values ranging approximately from 0.40 to 0.80. The weakest correlation was observed with SSDE_center_, with *R* ^2^ values approximately between 0.35 and 0.75. Furthermore, linear regression equations between each organ dose and the three SSDE metrics were derived. The slopes of these equations provided the estimation coefficients for calculating organ dose from the respective radiation dose metrics. These results are presented in Figures 2, 3, 4, 5, 6, 7, 8.

**FIGURE 2:**
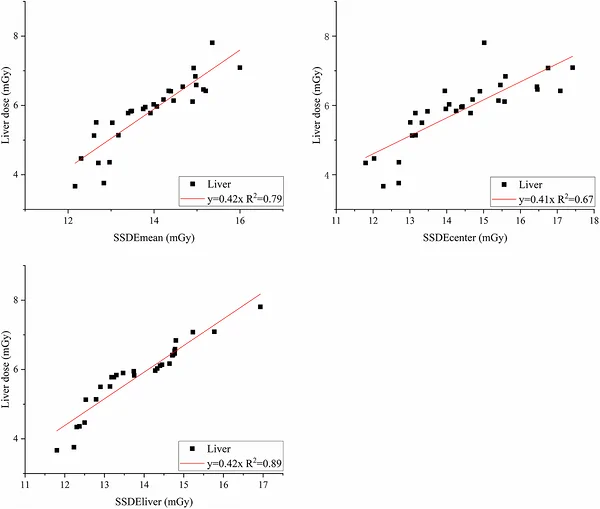
Linear fitting results between liver organ dose and the three SSDE metrics.

**FIGURE 3:**
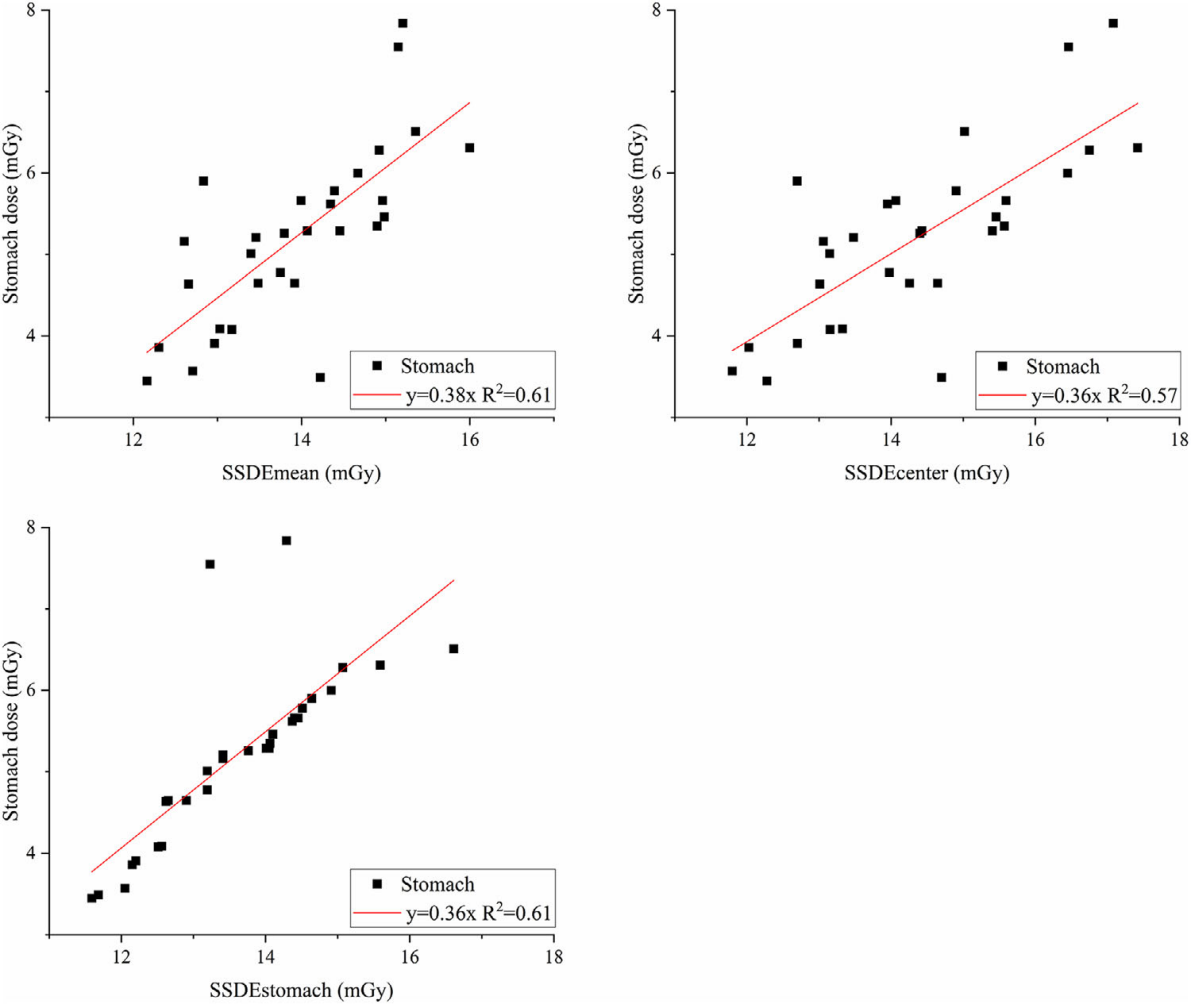
Linear fitting results between stomach organ dose and the three SSDE metrics.

**FIGURE 4:**
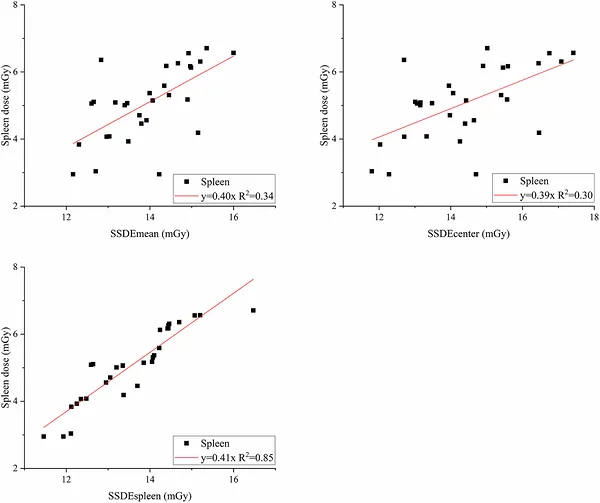
Linear fitting results between spleen organ dose and the three SSDE metrics.

**FIGURE 5:**
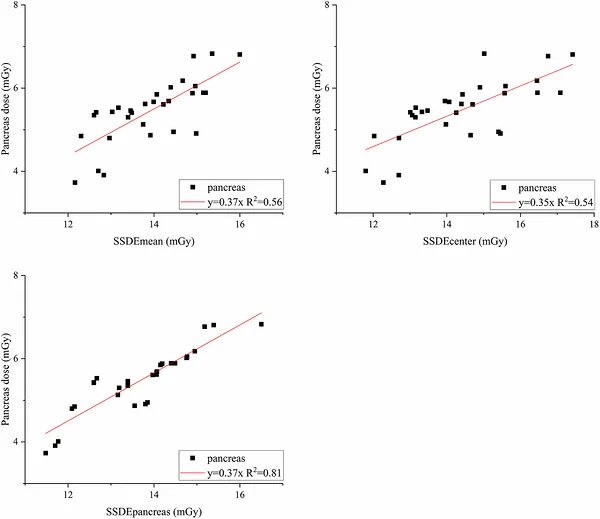
Linear fitting results between pancreas organ dose and the three SSDE metrics.

**FIGURE 6:**
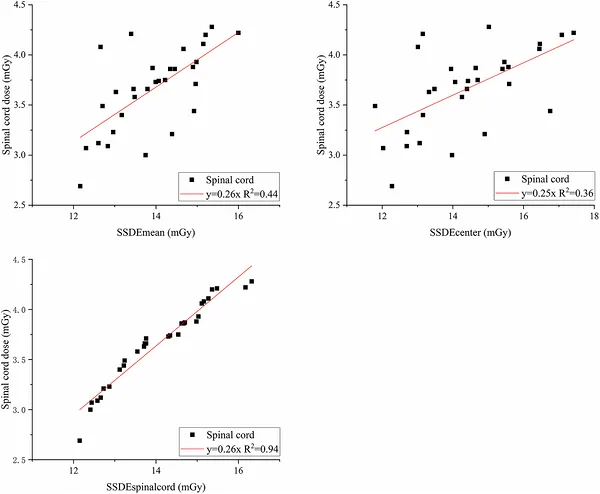
Linear fitting results between spinal cord organ dose and the three SSDE metrics.

**FIGURE 7:**
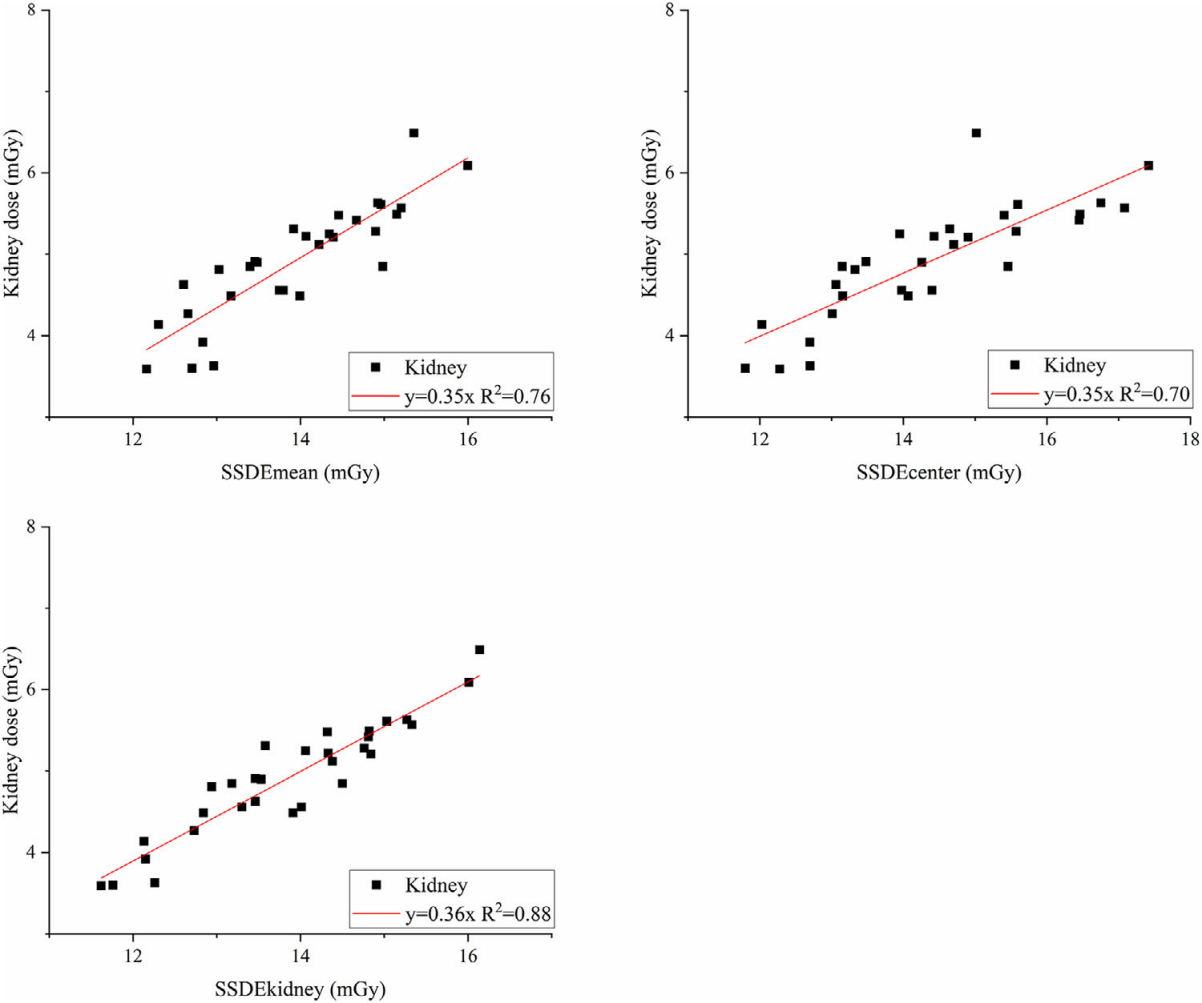
Linear fitting results between kidney organ dose and the three SSDE metrics.

**FIGURE 8:**
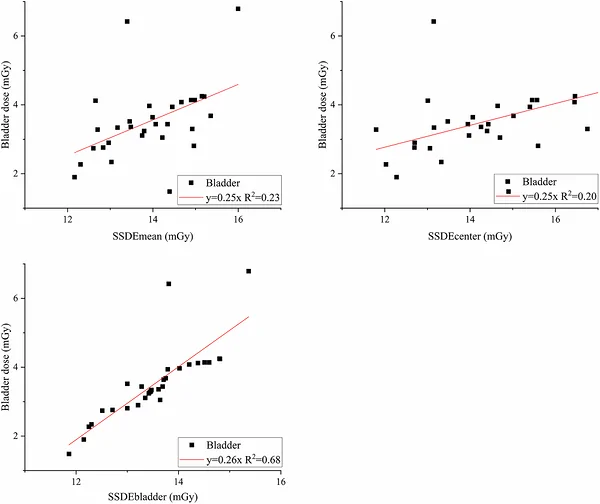
Linear fitting results between bladder organ dose and the three SSDE metrics.

To provide specific error data for clinical reference, this study calculated the mean absolute percentage error (MAPE) and its range for the organ doses estimated by SSDE_organ_ using the conversion coefficient, relative to the organ doses from MC simulations. In addition, the relative deviations and their ranges of the raw SSDE_mean_ and SSDE_center_ values from the organ doses were also computed (since these two metrics exhibit low correlation with organ dose, making the use of the conversion coefficient unreliable). Detailed data are presented in Table S2 in the Supplementary Materials. The results showed that the estimation errors for SSDE_organ_ were favorable in solid organs (liver 7.34%, kidney 6.09%, spleen 13.25%, spinal cord 2.97%, pancreas 13.61%), whereas relatively larger errors were observed in hollow organs such as the stomach (17.64%) and bladder (18.56%). Moreover, the deviations of SSDE_mean_ and SSDE_center_ from the organ doses were approximately 200%–300%.

## 4 DISCUSSION

MC simulation is widely regarded as the “gold standard” for calculating organ doses to patients in CT examinations.^34^ However, the clinical translation of MC simulations is limited by three factors: they are time‐consuming, require high operator expertise, and demand substantial hardware capability. This study analyzes the relationship between organ doses and three dose metrics—SSDE_mean_, SSDE_center_, and SSDE_organ_ in patients undergoing abdominal spectral CT examinations. In the following discussion, all references to “organ dose” refer to the MC‐simulated values described in Section 2.5.2. The aim is to establish a rapid method for estimating organ dose, thereby overcoming the time‐consuming and technically demanding limitations associated with MC simulations. The results indicate that all three metrics show significant correlations with organ dose, with SSDE_organ_ exhibiting the highest correlation. Therefore, in abdominal spectral CT examinations, employing SSDE_organ_ enables more accurate and efficient estimation of organ dose. This research holds significant implications for advancing prospective, patient‐specific radiation dose monitoring and optimizing CT scanning protocols.

Due to the varying radiation sensitivity of different organs, coupled with inter‐patient size differences and potential intra‐patient size variations within the scan range, using CTDI_vol_ to assess individual organ dose is challenging.^35^ Previous studies have shown that compared to CTDI_vol_, SSDE provides a more accurate representation of the actual radiation dose received by patients.^36^ This study incorporated three SSDE metrics for correlation analysis with organ dose. Figures 2, 3, 4, 5, 6, 7, 8 presents the linear fitting results and *R* ^2^ values for the correlation between organ dose and the three SSDEs for seven radiation‐sensitive organs. The results show that the *R* ^2^ values between the three SSDEs and organ dose, ranked from highest to lowest, are: SSDE_organ_, SSDE_mean_, and SSDE_center_. Therefore, SSDE_organ_ is the preferred metric for rapid and accurate estimation of individual organ dose. Previous studies based on conventional SECT have confirmed that SSDE_center_, calculated based on a single central slice image, serves as a practical and efficient alternative metric for assessing the overall SSDE across the scan range, with its primary application being rapid evaluation of total examination dose.^18^ However, whether this finding holds for spectral CT (DECT) remains unclear. To evaluate the performance of the metric for organ dose estimation in spectral CT, we compared it with SSDE_organ_, which is used for organ‐specific assessment, and with SSDE_mean_. Our experimental results demonstrate that, in estimating organ‐specific dose, SSDE_center_ exhibited the lowest correlation among the three metrics, while SSDE_mean_ showed moderate correlation. In contrast, SSDE_organ_ demonstrated the highest correlation, a finding consistent with that reported by Fujii et al.,^19^ in the context of conventional CT. The quantitative error analysis in Supplementary Table S2 further confirms that the MAPE of SSDE_organ_ estimates for all solid organs was below 14%, indicating clinically acceptable estimation accuracy. Therefore, despite the minimal difference between SSDE_center_ and SSDE_mean_ reported in SECT studies,^37^ our findings indicate that using SSDE_center_ to estimate organ dose in abdominal spectral CT has limited accuracy, highlighting the necessity of re‐evaluating these metrics specifically for DECT. Consequently, for rapid organ dose estimation in clinical practice, the SSDE_organ_ metric is recommended as the preferred choice.

The linear fitting results indicate the following estimation coefficients for SSDE_organ_ for each organ: liver: 0.42, stomach: 0.36, spleen: 0.41, pancreas: 0.37, spinal cord: 0.26, kidneys: 0.36, bladder: 0.26. These values differ considerably from the conversion coefficients (liver, 0.94; stomach, 0.92; bladder, 0.89) reported by Fujii et al.,^19^ in conventional CT (120 kV) studies. This discrepancy can be attributed to two primary factors. First, the present study simulated a rapid kV‐switching spectral CT scanning mode, in which the total mAs is equally divided between 80 and 140 kV. The introduction of low‑energy components alters the X‑ray energy spectrum distribution, resulting in organ dose profiles that are markedly different from those in conventional SECT.^38^, ^39^ Second, inherent differences in the X‑ray energy spectra among different CT scanners may also affect the final MC simulation outcomes. It is noteworthy that, in spectral CT scans, the values of all three SSDE metrics (SSDE_center_, SSDE_mean_, and SSDE_organ_) were systematically higher than the MC‐simulated organ doses. Previous conventional CT studies have shown that the discrepancy between SSDE and organ dose can be controlled within 10%^40^, ^41^; however, in this study, we found that the SSDE values in spectral CT were typically 200%‐300% higher than the organ doses, with a far greater difference than that observed in conventional CT. This phenomenon is further supported by ionization chamber measurements: under identical 100 mAs conditions, the pencil ionization chamber reading was measured as 10.75 mGy for the 100/140 kV spectral scan mode, whereas for the conventional 120 kV CT mode it was measured as 20.7 mGy,^13^ indicating that organ dose levels in spectral CT are substantially lower, which consequently further amplifies the relative deviation between SSDE and organ dose. Previous studies have indicated that even in conventional CT, the ± 20% deviation range between SSDE and organ dose is insufficient to encompass 95% of individual patients (i.e., more than 5% of patients exhibit deviations exceeding ± 20%), indicating inherent limitations of SSDE as a surrogate for organ dose.^42^, ^43^, ^44^ To address this, the present study established a linear regression model between SSDE_organ_ and organ dose, thereby providing a personalized organ dose estimation method for abdominal spectral CT that balances simplicity and accuracy. Furthermore, the data analysis revealed that the morphological and positional stability of the organs themselves significantly influenced the fitting performance. For solid organs such as the liver, kidneys, and spinal cord, which have relatively fixed morphologies, the R^2^ between organ dose and SSDE_organ_ was all above 0.80. In contrast, for hollow organs such as the stomach and bladder, which are subject to variations in filling status and consequently exhibit greater fluctuations in delineated volumes, the MC‐simulated organ dose showed abnormally elevated values, resulting in lower correlations (*R* ^2^ = 0.61 and 0.68, respectively. Therefore, attention should also be paid to the variability when using SSDE_organ_ to estimate doses for hollow organs.

This study has several limitations. First, due to computational constraints, MC simulations were performed on only 30 patients. Second, the bowtie filter model is simplified and may not perfectly replicate the actual geometry. Third, our dose validation relied on homogeneous‐phantom CTDIw measurements at single‐energy settings, and we used linear superposition to infer spectral‐mode doses; however, direct experimental verification under actual spectral scanning conditions was not performed. Fourth, our voxelized models were constructed exclusively from slices within the scan range, so scattered photons from outside the scan range were not accounted for, potentially leading to systematic underestimation of organ doses. In addition, the organ doses reported herein represent only the exposed portion of each organ, rather than the whole‐organ mean dose. Finally, the proposed method is specifically tailored to the spectral mode of the Revolution CT scanner, limiting its generalizability to other spectral CT platforms. Future work should address these gaps through direct physical measurements using anthropomorphic phantoms in spectral mode and extend the validation to a broader range of scanner types.

## 5 CONCLUSION

The results of this study indicate that for abdominal spectral CT examinations, organ doses for the liver, stomach, pancreas, spleen, kidneys, spinal cord, and bladder exhibit the highest correlation with SSDE_organ_, which is stronger than with SSDE_center_ and SSDE_mean_, with the corresponding estimation errors falling within acceptable ranges. Therefore, the linear regression fit between organ dose and SSDE_organ_ will facilitate rapid estimation of individualized organ doses in abdominal spectral CT. It is noteworthy, however, that the estimation factors for spectral CT differ from those for conventional CT examinations.

## AUTHOR CONTRIBUTIONS

**Tian Qin**: writing—original draft, supervision, methodology, investigation, formal analysis, data curation. **Qingting Sun**: writing—review and editing. **Yihan Fan**: writing—review and editing. **Mengting Wang**: writing—review and editing. **Jing Wang**: writing—review and editing. **Yankui Chang**: writing—review and editing. **Baohui Liang**: writing—review and editing, supervision, project administration, methodology, funding acquisition, conceptualization.

## FUNDING INFORMATION

The authors have nothing to report.

## CONFLICT OF INTEREST STATEMENT

The authors have no relevant conflicts of interest to disclose.

## ETHICAL APPROVAL

This study was approved by the Ethics Committee of Bengbu Medical University (NO.2025‐508). The study complied with the Declaration of Helsinki.

## Supporting information



Supporting Information

Supporting Information

## Data Availability

The data presented in this study are available on request from the corresponding author [Baohui Liang].

## References

[1] Forghani R , De Man B , Gupta R . Dual‐energy computed tomography: physical principles, approaches to scanning, usage, and implementation: part 1. Neuroimaging Clin N Am. 2017;27(3):371‐384. doi:10.1016/j.nic.2017.03.002 28711199

[2] Eliahou R , Hidas G , Duvdevani M , Sosna J . Determination of renal stone composition with dual‐energy computed tomography: an emerging application. Semin Ultrasound CT MR. 2010;31(4):315‐320. doi:10.1053/j.sult.2010.05.002 20691932

[3] Agrawal MD , Pinho DF , Kulkarni NM , Hahn PF , Guimaraes AR , Sahani DV . Oncologic applications of dual‐energy CT in the abdomen. Radiographics. 2014;34(3):589‐612. doi:10.1148/rg.343135041 24819783

[4] Huda W . Radiation risks: what is to be done?. AJR Am J Roentgenol. 2015;204(1):124‐127. doi:10.2214/AJR.14.12834 25539247

[5] Paul J , Banckwitz R , Krauss B , Vogl TJ , Maentele W , Bauer RW . Estimation and comparison of effective dose (E) in standard chest CT by organ dose measurements and dose‐length‐product methods and assessment of the influence of CT tube potential (energy dependency) on effective dose in a dual‐source CT. Eur J Radiol. 2012;81(4):e507‐12. doi:10.1016/j.ejrad.2011.06.006 21703793

[6] Henzler T , Fink C , Schoenberg SO , Schoepf UJ . Dual‐energy CT: radiation dose aspects. AJR Am J Roentgenol. 2012;199(supp l):S16‐25. doi:10.2214/AJR.12.9210 23097163

[7] Schenzle JC , Sommer WH , Neumaier K , et al. Dual energy CT of the chest: how about the dose?. Invest Radiol. 2010;45(6):347‐353. doi:10.1097/RLI.0b013e3181df901d 20404737

[8] Gu J , Bednarz B , Caracappa PF , Xu XG . The development, validation and application of a multi‐detector CT (MDCT) scanner model for assessing organ doses to the pregnant patient and the fetus using Monte Carlo simulations. Phys Med Biol. 2009;54(9):2699‐2717. doi:10.1088/0031-9155/54/9/007 PMC3376893 19351983

[9] Lee C , Liu J , Griffin K , Folio L , Summers RM . Adult patient‐specific CT organ dose estimations using automated segmentations and Monte Carlo simulations. Biomed Phys Eng Express. 2020;6(4):045016. doi:10.1088/2057-1976/ab98e6 33444276

[10] Dabin J , Mencarelli A , McMillan D , Romanyukha A , Struelens L , Lee C . Validation of calculation algorithms for organ doses in CT by measurements on a 5 year old paediatric phantom. Phys Med Biol. 2016;61(11):4168‐4182. doi:10.1088/0031-9155/61/11/4168 27192093

[11] DeMarco JJ , Cagnon CH , Cody DD , et al. A Monte Carlo based method to estimate radiation dose from multidetector CT (MDCT): cylindrical and anthropomorphic phantoms. Phys Med Biol. 2005;50(17):3989‐4004. doi:10.1088/0031-9155/50/17/005 16177525

[12] Shdeed TA , Nahili M , Issa NA , Bitar AJ . Study of absorbed dose in important organs during helical CT chest scan using MCNP code and MIRD phantom. Egypt J Radiol Nucl Med. 2016;47:1649‐1663.

[13] Pakravan D , Babapour Mofrad F , Deevband MR , Ghorbani M , Pouraliakbar H . Organ dose in cardiac dual‐energy computed tomography: a Monte Carlo study. Phys Eng Sci Med. 2022;45(1):157‐166. doi:10.1007/s13246-021-01098-9 35015205

[14] Pakravan D . Presentation of organ dose and effective dose conversion factors in dual‐energy computed tomography: a Monte Carlo simulation study. J Biomed Phys Eng. 2023;13(4):333‐344. doi:10.31661/jbpe.v0i0.2301-1586 PMC10440407 37609513

[15] Zhao J , Chen YL , Yang ZY , Xi Y . Investigation of dose for the triple‐source computed tomography based on Monte Carlo simulations. J Imaging Sci. 2016;1(1):1‐7. doi:10.15436/2381-0793.16.1142

[16] AAPM . Size‐specific dose estimates (SSDE) in pediatric and adult body CT examinations. AAPM Report No.204. American Association of Physicists in Medicine. 2011. http://www.aapm.org/pubs/reports/RPT_204.pdf

[17] AAPM . Use of water equivalent diameter for calculating patient size and size‐specific dose estimates (SSDE) in CT. AAPM Report No.220. American Association of Physicists in Medicine. 2014. https://www.aapm.org/pubs/reports/RPT_220.pdf PMC4991550 27546949

[18] Leng S , Shiung M , Duan X , Yu L , Zhang Y , McCollough CH . Size‐specific dose estimates for chest, abdominal, and pelvic CT: effect of intrapatient variability in water‐equivalent diameter. Radiology. 2015;277(1):308‐309. doi:10.1148/radiol.2015151209 10.1148/radiol.2015151209 PMC7006770 26402502

[19] Fujii K , Nomura K , Muramatsu Y , et al. Correlation analysis of organ doses determined by Monte Carlo simulation with dose metrics for patients undergoing chest‐abdomen‐pelvis CT examinations. Physica Med. 2020;77:1‐9. doi:10.1016/j.ejmp.2020.07.016 10.1016/j.ejmp.2020.07.016 32755745

[20] Shohji T , Kuriyama K , Yanano N , Maeda E , Katoh Y . Simple method of measuring SSDE for head CT: facilitating pre‐CT scan dose calculation using specialized head scan band. Radiat Prot Dosimetry. 2021;197(1):1‐11. doi:10.1093/rpd/ncab149 10.1093/rpd/ncab149 34718785

[21] Abuhaimed A , Martin CJ . Effect of X‐ray spectrum on size‐specific dose estimates (SSDEs) for paediatric and adult patients undergoing CT scans. Radiat Phys Chem. 2021;179:109202. doi:10.1016/j.radphyschem.2020.109202

[22] Euler A , Obmann MM , Szucs‐Farkas Z , et al. Comparison of image quality and radiation dose between split‐filter dual‐energy images and single‐energy images in single‐source abdominal CT. Eur Radiol. 2018;28(8):3405‐3412. doi:10.1007/s00330‐018‐5338‐x 10.1007/s00330-018-5338-x 29460070

[23] Machida H , Tanaka I , Fukui R , et al. Dual‐energy spectral CT: various clinical vascular applications. Radiographics. 2016;36(4):1215‐1232. doi:10.1148/rg.2016150185 10.1148/rg.2016150185 27399244

[24] ICRP . Adult reference computational phantoms. ICRP publication 110. Ann ICRP. 2009;39(2):1‐166. doi:10.1016/j.icrp.2009.09.001 10.1016/j.icrp.2009.09.001 19897132

[25] Turner AC , Zhang D , Khatonabadi M , et al. The feasibility of patient size‐corrected, scanner‐independent organ dose estimates for abdominal CT exams. Med Phys. 2011;38(2):820‐829. doi:10.1118/1.3533897 10.1118/1.3533897 PMC3037972 21452719

[26] Pan Y , Qiu R , Gao L , et al. Development of 1‐year‐old computational phantom and calculation of organ doses during CT scans using Monte Carlo simulation. Phys Med Biol. 2014;59(18):5243‐5260. doi:10.1088/0031‐9155/59/18/5243 10.1088/0031-9155/59/18/5243 25144385

[27] Yang K , Li X , Liu B . Direct and fast measurement of CT beam filter profiles with simultaneous geometrical calibration. Med Phys. 2017;44(1):57‐70. doi:10.1002/mp.12024 10.1002/mp.12024 PMC5543987 28102951

[28] Khursheed A , Hillier MC , Shrimpton PC , Wall BF . Influence of patient age on normalized effective doses calculated for CT examinations. Br J Radiol. 2022;75(898):819‐830. doi:10.1259/bjr.75.898.750819 10.1259/bjr.75.898.750819 12381691

[29] Goo HW , Goo JM . Dual‐Energy CT: new horizon in medical imaging. Korean J Radiol. 2017;18(4):555‐569. doi:10.3348/kjr.2017.18.4.555 10.3348/kjr.2017.18.4.555 PMC5447632 28670151

[30] Marin D , Boll DT , Mileto A , Nelson RC . State of the art: dual‐energy CT of the abdomen. Radiology. 2014;271(2):327‐342. doi:10.1148/radiol.14131480 10.1148/radiol.14131480 24761954

[31] Greffier J , Viry A , Barbotteau Y , et al. Phantom task‐based image quality assessment of three generations of rapid kV‐switching dual‐energy CT systems on virtual monoenergetic images. Med Phys. 2022;49(4):2233‐2244. doi:10.1002/mp.15558 10.1002/mp.15558 35184293

[32] Jung DC , Oh YT , Kim MD , Park M . Usefulness of the virtual monochromatic image in dual‐energy spectral CT for decreasing renal cyst pseudoenhancement: a phantom study. AJR Am J Roentgenol. 2012;199(6):1316‐1319. doi:10.2214/AJR.12.8660 10.2214/AJR.12.8660 23169724

[33] Matsumoto K , Jinzaki M , Tanami Y , Ueno A , Yamada M , Kuribayashi S . Virtual monochromatic spectral imaging with fast kilovoltage switching: improved image quality as compared with that obtained with conventional 120‐kVp CT. Radiology. 2011;259(1):257‐262. doi:10.1148/radiol.11100978 10.1148/radiol.11100978 21330561

[34] Dosimetry DamilakisJCT . What has been achieved and what remains to be done. Invest Radiol. 2021;56(1):62‐68. doi:10.1097/RLI.0000000000000727 10.1097/RLI.0000000000000727 32932380

[35] Shope TB , Gagne RM , Johnson GC . A method for describing the doses delivered by transmission x‐ray computed tomography. Med Phys. 1981;8(4):488‐495. doi:10.1118/1.594995 10.1118/1.594995 7322067

[36] He J , Dong G , Deng Y , He J , Xiu Z , Feng F . Comparison of application value of different radiation dose evaluation methods in evaluating radiation dose of adult thoracic and abdominal CT Scan. Front Surg. 2022;9:860968. doi:10.3389/fsurg.2022.860968 10.3389/fsurg.2022.860968 PMC8990916 35402481

[37] Cheng PM . Automated estimation of abdominal effective diameter for body size normalization of CT dose. J Digit Imaging. 2013;26(3):406‐411. doi:10.1007/s10278‐012‐9525‐z 10.1007/s10278-012-9525-z PMC3649058 22955797

[38] Vaniqui A , Schyns L , Almeida IP , van der Heyden B , van Hoof SJ , Verhaegen F . The impact of dual energy CT imaging on dose calculations for pre‐clinical studies. Radiat Oncol. 2017;12(1):181. doi:10.1186/s13014‐017‐0922‐9 10.1186/s13014-017-0922-9 PMC5696722 29157265

[39] Mahmood U , Horvat N , Horvat JV , et al. Rapid switching kVp dual energy CT: value of reconstructed dual energy CT images and organ dose assessment in multiphasic liver CT exams. Eur J Radiol. 2018;102:102‐108. doi:10.1016/j.ejrad.2018.02.022 10.1016/j.ejrad.2018.02.022 PMC5918634 29685522

[40] Turner AC , Zankl M , DeMarco JJ , et al. The feasibility of a scanner‐independent technique to estimate organ dose from MDCT scans: using CTDIvol to account for differences between scanners. Med Phys. 2010;37(4):1816‐1825. doi:10.1118/1.3368596 10.1118/1.3368596 PMC2861967 20443504

[41] Moore BM , Brady SL , Mirro AE , Kaufman RA . Size‐specific dose estimate (SSDE) provides a simple method to calculate organ dose for pediatric CT examinations. Med Phys. 2014;41(7):071917. doi:10.1118/1.4884227 10.1118/1.4884227 PMC5148074 24989395

[42] Hardy AJ , Bostani M , Kim GHJ , Cagnon CH , Zankl MA , McNitt‐Gray M . Evaluating size‐specific dose estimate (SSDE) as an estimate of organ doses from routine CT exams derived from Monte Carlo simulations. Med Phys. 2021;48(10):6160‐6173. doi:10.1002/mp.15128 10.1002/mp.15128 34309040

[43] Li X , Marschall TA , Yang K , Liu B . Technical note: advancing size‐specific dose estimates in CT examinations: dose estimates at longitudinal positions of scans. Med Phys. 2022;49(2):1303‐1311. doi:10.1002/mp.15402 10.1002/mp.15402 34894408

[44] Abdulkadir MK , Shuaib IL , Achuthan A , Nasirudin RA , Samsudin AHZ , Osman ND . Estimation of pediatric dose descriptors adapted to individual specific size from CT examinations. Radiat Prot Dosim. 2022;198(17):1292‐1302. doi:10.1093/rpd/ncac163 10.1093/rpd/ncac163 35896148

