# Supplementary material for: Correlation of size specific dose estimates with organ dose in abdominal spectral computed tomography
Source: J Appl Clin Med Phys. 2026 Oct 5;27(10):e70845. doi: 10.1002/acm2.70845 (PMC13639253; doi:10.1002/acm2.70845)
Supplement: Supplementary file 1 [file ACM2-27-e70845-s001.doc]

Table S-2: Estimation error of SSDEorgan and related deviations of SSDEmean and SSDEcenter (Mean ± SD)

| Organ | SSDEorgan (%) | SSDEmean (%) | SSDEcenter (%) |
| --- | --- | --- | --- |
| Liver | 7.34(9.33) | 143.51(32.65) | 150.20(29.53) |
| Stomach | 17.64(10.23) | 176.00(46.85) | 183.35(43.91) |
| Spleen | 13.25(12.73) | 187.22(63.81) | 195.19(63.36) |
| Pancreas | 13.61(9.51) | 158.40(28.87) | 165.74(27.74) |
| Spinal cord | 2.97(3.41) | 283.63(33.87) | 295.21(39.55) |
| Kidney | 6.09(4.30) | 186.98(27.93) | 194.97(23.98) |
| Bladder | 18.56(22.95) | 328.63(135.21) | 340.80(138.43) |
