# Supplementary material for: Correlation of size specific dose estimates with organ dose in abdominal spectral computed tomography
Source: J Appl Clin Med Phys. 2026 Oct 5;27(10):e70845. doi: 10.1002/acm2.70845 (PMC13639253; doi:10.1002/acm2.70845)
Supplement: Supplementary file 2 [file ACM2-27-e70845-s002.doc]

Table S-1: Comparison of simulated and measured CTDI100 in the central and peripheral holes under different tube voltages and collimation widths.

| Tube voltage  (kV) | Collimation  (mm) | Position | CTDI_100, M_  (mGy/100 mAs) | CTDI_100, S_  (mGy/100 mAs) | Error (100%) |
| --- | --- | --- | --- | --- | --- |
| 80 | 40 | Center | 1.04 | 0.95 | -7.88% |
|  |  | Peripheral | 2.66 | 2.46 | -7.48% |
|  | 80 | Center | 0.94 | 0.86 | -9.55% |
|  |  | Peripheral | 2.35 | 2.20 | -6.38% |
| 100 | 40 | Center | 2.28 | 2.18 | -4.71% |
|  |  | Peripheral | 5.04 | 4.72 | -6.38% |
|  | 80 | Center | 2.09 | 1.95 | -6.45% |
|  |  | Peripheral | 4.52 | 4.29 | -5.15% |
| 120 | 40 | Center | 3.95 | 3.84 | -2.77% |
|  |  | Peripheral | 8.03 | 7.53 | -6.23% |
|  | 80 | Center | 3.60 | 3.47 | -3.69% |
|  |  | Peripheral | 7.14 | 6.81 | -4.53% |
| 140 | 40 | Center | 5.94 | 5.71 | -3.92% |
|  |  | Peripheral | 11.33 | 1.07 | -5.20% |
|  | 80 | Center | 5.41 | 5.16 | -4.75% |
|  |  | Peripheral | 10.06 | 9.59 | -4.69% |
